# Austrian consensus on the diagnosis and management of portal hypertension in advanced chronic liver disease (Billroth IV)

**DOI:** 10.1007/s00508-023-02229-w

**Published:** 2023-06-26

**Authors:** Mattias Mandorfer, Elmar Aigner, Manfred Cejna, Arnulf Ferlitsch, Christian Datz, Tilmann Gräter, Ivo Graziadei, Michael Gschwantler, Stephanie Hametner-Schreil, Harald Hofer, Mathias Jachs, Alexander Loizides, Andreas Maieron, Markus Peck-Radosavljevic, Florian Rainer, Bernhard Scheiner, Georg Semmler, Lukas Reider, Silvia Reiter, Maria Schoder, Rainer Schöfl, Philipp Schwabl, Vanessa Stadlbauer, Rudolf Stauber, Elisabeth Tatscher, Michael Trauner, Alexander Ziachehabi, Heinz Zoller, Peter Fickert, Thomas Reiberger

**Affiliations:** 1 Division of Gastroenterology and Hepatology, Department of Internal Medicine III, Medical University of Vienna, Währinger Gürtel 18–20, 1090 Vienna, Austria; 2 Vienna Hepatic Hemodynamic Laboratory, Division of Gastroenterology and Hepatology, Department of Internal Medicine III, Medical University of Vienna, Vienna, Austria; 3 First Department of Medicine, Paracelsus Medical University, Salzburg, Austria; 4 Department of Radiology, LKH Feldkirch, Feldkirch, Austria; 5 Department of Internal Medicine I, KH Barmherzige Brüder Wien, Vienna, Austria; 6 Department of Internal Medicine, General Hospital Oberndorf, Teaching Hospital of the Paracelsus Medical University Salzburg, Salzburg, Austria; 7 Department of Radiology, Medical University of Graz, Graz, Austria; 8 Department of Internal Medicine, KH Hall in Tirol, Hall, Austria; 9 Division of Gastroenterology and Hepatology, Department of Medicine IV, Klinik Ottakring, Vienna, Austria; 10 Department of Gastroenterology and Hepatology, Ordensklinikum Linz Barmherzige Schwestern, Linz, Austria; 11 Department of Internal Medicine I, Klinikum Wels-Grieskirchen, Wels, Austria; 12 Department of Radiology, Medical University of Innbsruck, Innsbruck, Austria; 13 Department of Internal Medicine II, University Hospital St. Pölten, St. Pölten, Austria; 14 Department of Internal Medicine and Gastroenterology, Hepatology, Endocrinology, Rheumatology and Nephrology, Klinikum Klagenfurt am Wörthersee, Klagenfurt, Austria; 15 Division of Gastroenterology and Hepatology, Department of Internal Medicine, Medical University of Graz, Graz, Austria; 16 Department of Interventional Radiology, Medical University of Vienna, Vienna, Austria; 17 Department of Internal Medicine and Gastroenterology and Hepatology, Kepler Universitätsklinikum, Linz, Austria; 18 Department of Internal Medicine I, Medical University of Innsbruck, Innsbruck, Austria

**Keywords:** Cirrhosis, Elastography, HVPG, Varices, Variceal bleeding, Acute-on-chronic liver failure, Ascites, Spontaneous bacterial peritonitis, Hepatorenal syndrome, Transjugular intrahepatic portosystemic shunt, Portal vein thrombosis

## Abstract

The Billroth IV consensus was developed during a consensus meeting of the Austrian Society of Gastroenterology and Hepatology (ÖGGH) and the Austrian Society of Interventional Radiology (ÖGIR) held on the 26th of November 2022 in Vienna.

Based on international recommendations and considering recent landmark studies, the Billroth IV consensus provides guidance regarding the diagnosis and management of portal hypertension in advanced chronic liver disease.

## Grading of certainty and recommendation

**Certainty in evidence** was determined in analogy to the GRADE framework [1], as also applied by the Baveno VII consensus [2]:

- Very low (**D**): The true effect is probably markedly different from the estimated effect.
- Low (**C**): The true effect might be markedly different from the estimated effect.
- Moderate (**B**): The authors believe that the true effect is probably close to the estimated effect.
- High (**A**): The authors have a lot of confidence that the true effect is similar to the estimated effect.

Classifications that have been endorsed by major societies, are broadly accepted, and unlikely to see major changes until the next edition of the Billroth recommendations have generally been graded as B1, although the GRADE system has limited applicability in this context. Notably, the certainty in the evidence has been be rated up in some occasions (usually when there is a very large magnitude of effect, e.g., transplant benefit in patients with refractory ascites), as the conduct of high-quality trials would be considered unethical, since a meaningful treatment benefit is almost certain from observational studies.

Similarly, GRADE framework was applied to provide a **strength of recommendation**:

- Weak (**2**): Indicates that engaging in a shared decision-making process is essential.
- Strong (**1**): Suggests that it is usually necessary to present both options.

## 1. Definition, diagnosis, work-up and follow-up of compensated advanced chronic liver disease (cACLD)

### Definition of cACLD

- The term compensated advanced chronic liver diseases (cACLD) describes a spectrum of advanced fibrosis and cirrhosis (i.e., F3/F4) with or without associated portal hypertension in patients with an ongoing (i.e., unresolved) primary aetiological factor and in the absence of previous/current hepatic decompensation (i.e., ascites grade ≥ 2, variceal bleeding, or overt hepatic encephalopathy) [2]. (B1)
- Although patients in whom the primary aetiological factor has been removed are formally excluded from the definition of cACLD due to differences in regard to non-invasive tests (NIT) and risk stratification, those with findings that are compatible with cACLD should be managed similarly, unless specified otherwise or until further evidence becomes available. (C1)
- Both ‘cACLD’ and ‘compensated cirrhosis’ are applicable, but not equivalent. The term cACLD describes patients at increased risk for liver-related events and considers that their identification/the diagnosis of cACLD primarily relies on NIT. In contrast, compensated cirrhosis dates back to pre-NIT era, when it was diagnosed by histology or less sensitive imaging and laboratory criteria. (B1)

### Diagnosis of cACLD

- NIT have to be interpreted in the light of potential confounding factors (e.g., biochemical evidence of hepatic inflammation (AST, ALT, or GGT > 2 × upper normal limit [3]), extrahepatic cholestasis, congestion, and food intake increase liver stiffness measurement (LSM) [4]; systemic inflammation and extrahepatic fibrotic diseases increase ELF test), which may lead to false-positive results [4]. (B1)
- The cornerstone in the diagnosis of cACLD is LSM by vibration-controlled transient elastography (VCTE). LSM values < 10 kPa rule-out cACLD, LSM values of 10–15 kPa are suggestive of cACLD, while LSM values ≥ 15 kPa are highly suggestive of cACLD. (B1)
- Due to potential false-positive results of VCTE, LSM should be repeated in fasting condition in those with ≥ 10 kPa. (B1)
- If VCTE is not available, alternative NIT for diagnosing cACLD may be applied (Table 1). A FIB‑4 score of 1.75 approximates a LSM by VCTE of 10 kPa and FIB‑4 values < 1.75 rule-out cACLD, as these patients are at negligible risk for hepatic decompensation. (B2)

**Table 1:** Alternative (i.e., non-VCTE-based) methods for diagnosing cACLD and identifying cACLD patients with a low/high probability of CSPH. A multitude of additional methods is capable of diagnosing cACLD (i.e., F3/F4) with adequate accuracy, however, only broadly used blood-based NIT and elastography methods for which cut-offs for ruling-in/ruling-out (i.e., high sensitivity/negative predictive value and specificity/positive predictive value) CSPH are available are mentioned

Method	Proprietary name/manufacturer	Strength/limitations	Cut-offs
**Diagnosis of cACLD**			
*LSM by 2D-SWE*	Aixplorer/Supersonic Imagine/HOLOGIC	Confounding factors are similar to those for VCTE provided in **Chap. 1**	Similar cut-offs as for VCTE
*LSM by 2D-SWE*	LOGIQ 2D Shear Wave Elastography/General Electric	Limited studies with liver biopsy as reference standard; Confounding factors are similar to those for VCTE provided in **Chap. 1**	> 9.3 kPa [237]
*FIB‑4 score*	Non-proprietary	No dedicated hard-/software; Lower diagnostic but similar prognostic performance vs. VCTE	≥ 1.75 [5]
*ELF test*	Siemens	Confounding factor provided in **Chap. 1**	≥ 9.8 [4, 238, 239]
**Identification of cACLD patients with a low/high probability of CSPH**			
*LSM by 2D-SWE*	Aixplorer/Supersonic Imagine/HOLOGIC	Most well-studied elastography method besides VCTE; Majority of studies not restricted to cACLD; Confounding factors are similar to those for VCTE provided in **Chap. 1**	Similar cut-offs/decision rules as for VCTE
*LSM by 2D-SWE*	LOGIQ 2D Shear Wave Elastography/General Electric	Single study [240] with a small cACLD subgroup; Confounding factors are similar to those for VCTE provided in the **Chap. 1**	CSPH ruled-out: < 9 kPa; CSPH ruled-in: > 13 kPa
*VITRO*	Non-proprietary	No dedicated hard-/software; Confounding factors are provided in **Chap. 1**	CSPH ruled-out: < 1 [5]; CSPH ruled-in: > 2.5 [5]

### Work-up and follow-up of patients with cACLD, or without

- Patients with cACLD should be referred to a specialized liver unit for individualized work-up and management. (B1)
- cACLD patients are at risk of hepatic decompensation, which is primarily driven by severity of portal hypertension. Diagnosis of cACLD should prompt an evaluation for clinically significant portal hypertension (CSPH), as the presence of CSPH **(i)** identifies the subgroup of patients who are at significant risk for hepatic decompensation and **(ii)** has important therapeutic implications [4]. (B1)
- In patients with cACLD, LSM (or, if unavailable, von Willebrand factor antigen (VWF; %) to platelet count (PLT; G/L) ratio (VITRO) [5]) may be repeated every 12 months to monitor disease progression or regression. (B2)
- Patients without cACLD, but with ongoing chronic liver disease should be monitored for progression to cACLD. (B1)
- Overweight/obesity, diabetes, and alcohol consumption contribute to liver disease progression as potentially modifiable cofactors and should always be addressed [2]. (B1)
- Statin use is safe in patients with cACLD [6]. (B1)

## 2. Measurement of the hepatic venous pressure gradient

- The hepatic venous pressure gradient (HVPG) is the gold standard to indirectly estimate the portal pressure gradient via minimally invasive catheterization of the hepatic vein. Right-heart catheterization and/or transjugular liver biopsy can be performed within the same procedure, if required [7, 8]. (B1)
- HVPG is calculated by subtracting the free hepatic venous pressure (FHVP) from the wedged hepatic venous pressure (WHVP) [8]. (A1)
- Cannulation via the right jugular vein may be preferable due to the angle of the hepatic vein junction, in particular if transjugular liver biopsy is planned [8]. (C1)
- Use of a pre-bent balloon occlusion catheter is preferred due to a superior cannulation rate of the hepatic vein and a superior occlusion capacity, as compared to conventional end-hole catheters without a balloon [9–11]. (B1)
- Fluoroscopic guidance is essential for introducing the occlusion catheter from the (preferably right) internal jugular vein into a large hepatic vein. Documentation of catheter positioning is recommended, as this may help to increase the reproducibility of measurements [8]. (B1)
- A real-time pressure recording system is mandatory for appropriate documentation and interpretation of the measured data. Zeroing should be performed prior to measurement and the tracings should be recorded at slow speed. The pressure transducer should be positioned at the cardiac level of the patient in supine position [8, 12]. (B1)
- Performing the procedure in an awake patient is recommended, since abdominal press, inspiration or expiration may facilitate the cannulation of the internal jugular and hepatic vein. Yet, if necessary, a low dose of midazolam (≤ 0.02 mg/kg body weight) can be administered without affecting hepatic hemodynamics [13]. Notably, use of propofol is **not** recommended as it affects HVPG [14, 15]. Although fentanyl at a dose of 1.0 or 1.5 μg/kg was safe and did not impact HVPG [16], its use during HVPG measurement—a generally well-tolerated procedure [17]—is **not** warranted as it may induce breathing artefacts. (B1)
- During the hemodynamic measurements, deep breathing should be avoided. Since food intake affects portal hemodynamics [18], the measurement should be performed in a fasted patient. (B1)
- Measurement of the WHVP should be performed in one of the three hepatic veins (usually the right or middle). In order to measure WHVP, the balloon should be expanded according to vessel size and optimal vascular occlusion should be confirmed with a small volume of contrast agent. In case of inappropriate occlusion or veno-venous communications, deeper insertion of the catheter should be evaluated. (B1)
- If veno-venous shunts prevent an appropriate occlusion, this must be reported, since it may result in an underestimation of WHVP and hint at the presence of porto-sinusoidal vascular disorder (PSVD) [19]. (B1)
- Recordings of WHVP should be for at least 60 s (or longer, if continuously increasing) to guarantee stable pressure readouts [12]. (C1)
- The FHVP should be measured 2–3 cm from the junction where the hepatic vein drains into the inferior vena cava [20]. (B1)
- Recordings of FHVP should be for at least 30 s to guarantee stable pressure readouts [12]. (C1)
- Pairs of WHVP and FHVP should be measured at least in triplicate. The final HVPG value constitutes the mean of three independent WHVP/FHVP measurement pairs. In case of inconsistent HVPG values after 3 measurements, possible sources of error (in particular, false-high FHVP values due to distal positioning and false-low WHVP due to insufficient wedging) have to be evaluated and measurements have to repeated until consistent HVPG values are reached. (B1)
- Measurement of the inferior vena cava pressure (IVCP) is mandatory. In case a pressure difference > 2 mm Hg between IVCP and FHVP is evident, presence of a post-hepatic venous obstruction (or catheter misplacement) should be investigated by contrast injection. In case of a pressure difference > 2 mm Hg between IVCP and FHVP, the HVPG has to be calculated by subtracting the inferior vena cava pressure (IVCP) from the wedged hepatic venous pressure (WHVP). (B1) [21]
- HVPG-measurements are indicated for risk stratification (in particular, if NIT are inconclusive) and monitoring the response to HVPG-lowering treatment [22, 23]. HVPG values > 5 mm Hg denote portal hypertension, while values ≥ 10 mm Hg indicate clinically significant portal hypertension (CSPH) [2]. (B1)
- Presence of CSPH is associated with an increased risk of post-hepatectomy liver failure, hepatic decompensation, and mortality in patients with hepatocellular carcinoma (HCC), in particular when undergoing major hepatectomy. [24, 25]. Moreover, the absence of CSPH identifies patients at low risk for hepatic decompensation, while HVPG values ≥ 16 and ≥ 20 mm Hg indicate a progressively increased risk of short-term mortality in patients undergoing extrahepatic abdominal surgery [26]. (B1)
- HVPG decreases to a value of < 12 mm Hg or reductions by ≥ 10–20% in response to acute and chronic NSBB treatment are associated with a reduced incidence of variceal bleeding or other decompensating events and a lower mortality [27–31]. (B1)
- In clinical trials focusing on the treatment of portal hypertension, HVPG dynamics are an excellent surrogate endpoint [27–32]. (B1)
- HVPG values reflect sinusoidal portal hypertension, and thus, may underestimate the severity of portal hypertension in pre- (e.g., portal [PVT] and splanchnic vein thrombosis) and post-hepatic (e.g., congestive hepatopathy) as well as pre-sinusoidal disorders, e.g., portosinosoidal vascular disorder (PSVD). (B1)
- Endoscopic ultrasound-guided measurement of the pressure in the portal and hepatic veins is usually performed under deep sedation [33], which is known to profoundly impact hepatic hemodynamics [14, 15]. Thus, the clinical utility of the portal pressure gradient (PPG) derived from endoscopic ultrasound-guided pressure measurements has yet to be established. (C2)

## 3. Non-invasive staging of portal hypertension in patients with cACLD/compensated cirrhosis

### General considerations

- HVPG-measurement remains the diagnostic gold standard, however, it requires considerable resources and expertise, which limits its applicability [8]. Thus, NIT may be applied to estimate the probability of CSPH in clinical practice. (B1)
- NIT for CSPH have to be interpreted in the context of potential confounding factors: While LSM-specific information is provided in **Chap. 1**, factors confounding the association between spleen stiffness measurement (SSM) as well as VITRO [23, 34–36] and HVPG are less well-studied. Notably, VWF increases in the context of infection [37]. (B1)
- LSM should be repeated in fasting condition before deriving therapeutic consequences [2, 4]. (B1)

### Ruling-out and ruling-in CSPH and indication for esophagogastroduodenoscopy

**Fig. 1:**
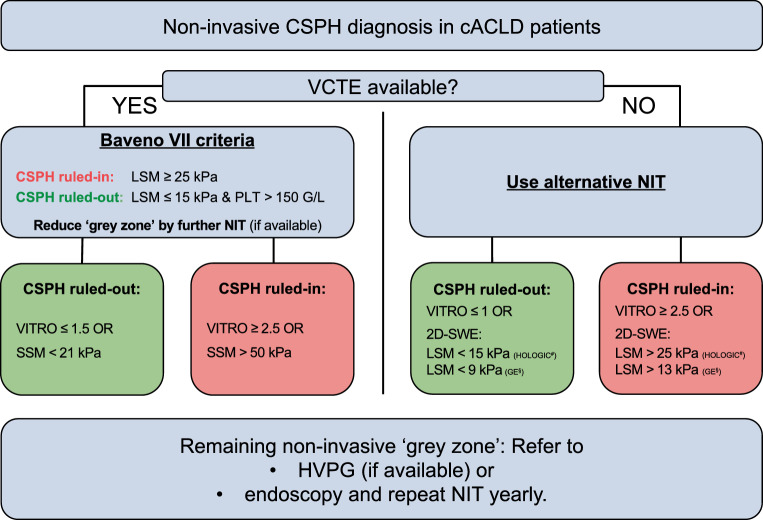
CSPH can be ruled-out or ruled-in by assessing LSM (by VCTE) and PLT and applying the Baveno VII criteria. Patients within the grey zone of the Baveno VII criteria may be reclassified by von Willebrand factor (VWF) to platelet count (PLT) ratio (VITRO) or spleen stiffness measurement SSM (by VCTE). Patients with inconclusive non-invasive findings should be evaluated by hepatic venous pressure gradient (HVPG)-measurement and/or endoscopy for the presence of clinically significant portal hypertension (CSPH) and varices (which are confirmative of CSPH), respectively. Alternative (i.e., non-VCTE-based) strategies, should limited to settings where VCTE is not available

- The Baveno VII criteria [2] for ruling-in/ruling-out CSPH should be applied (B1):
  - LSM by VCTE values ≤ 15 kPa & PLT ≥ 150 G/L rule-out CSPH (sensitivity and negative predictive value > 90%).
  - LSM by VCTE values ≥ 25 kPa rule-in CSPH (specificity and positive predictive value > 90%) in patients with viral hepatitis- and/or alcohol-related cACLD as well as non-obese non-alcoholic steatohepatitis (NASH).
- Those within the diagnostic grey zone of the above-mentioned criteria (i.e., meeting neither the Baveno VII rule-out nor rule-in criteria) can be re-classified by the additional consideration of either VITRO [38] or SSM ([39]; Fig. 1). (B1)
- If VCTE is not available, alternative NIT for identifying cACLD patients with a low/high probability of CSPH (e.g., VITRO [5]) may be applied, although the available evidence is more limited (Table 1). (B2)
- Patients with inconclusive non-invasive findings should be evaluated by HVPG-measurement and/or esophagogastroduodenoscopy (EGD) for the presence of CSPH and varices (which are confirmative of CSPH), respectively. (B1)
- cACLD patients in whom CSPH can be ruled-out based on NIT or HVPG are **not** required to undergo EGD, unless there is a suspicion of an additional pre-hepatic, i.e., PVT and/or splanchnic vein thrombosis, or an intrahepatic pre-sinusoidal cause/component of portal hypertension. The latter should be suspected in patients with [19]: (B2)
  - Clinical conditions associated with PSVD, e.g., myeloproliferative neoplasms.
  - Exposure to drugs that have been linked to PSVD (e.g., antiretroviral therapies (didanosine and stavudine), azathioprine, or oxaliplatin [40]).
  - Histological findings of PSVD [19].
  - Disproportionally low LSM despite imaging/laboratory evidence of portal hypertension [41] or disproportionally high SSM [42].

### Specific patient populations

- In patients with NASH-related cACLD, the ANTICIPATE-NASH model (which considers BMI in addition to LSM/PLT) can be used to estimate the probability of CSPH [43] (B2).
- Removal/suppression of the primary aetiological factor, i.e., HCV-cure, HBV-suppression in the absence of HDV infection, and abstinence from alcohol, may ameliorate portal hypertension, thereby reducing the risk of hepatic decompensation [2]. The definition and impact of the removal/suppression of the primary aetiological factor in other aetiologies is less well established, which does not necessarily imply that the respective therapies (e.g., phlebotomy for heamochromatosis) are less effective in modifying the course of cACLD. (B1)
- Patients with pre-treatment cACLD who show consistent improvements to LSM values < 12 kPa and PLT > 150 G/L after HCV-cure can be discharged from further portal hypertension, but **not** HCC surveillance measures, if no co-factors are present, as the risks of CSPH and disease progression/hepatic decompensation are negligible [44]. (B2)

## 4. Endoscopic classifications and treatment

### Esophageal varices

- Esophageal varices (EV) should be graded as absent, small (< 5 mm of diameter), or large (≥ 5 mm) [45]. (B1)
- The presence of red spots signs should be reported for bleeding risk stratification [45]. (B1)
- The indications for endoscopic therapy (i.e., endoscopic variceal ligation (EVL)) are described in **Chap. 5, 6 and 7**.
- EVL should be performed every 2–4 weeks until eradication of large varices. Thereafter, an endoscopy should be performed after 6 months and then every 12 months[45]. (B1)

### Gastroesophageal and gastric varices

- The Sarin classification should be used for classification of gastric varices [45] (B1):
  - Gastroesophageal varices type 1 (GOV1; varices from the lesser curvature extending into the esophagus) and 2 (GOV2; varices of the fundus continuing into the esophagus) as well as
  - isolated gastric varices 1 (IGV1; varices in the fundus not extending over the cardia) and 2 (IGV2; varices in other parts of the stomach).
- Risk of bleeding from gastric varices depends on subtype (IGV1 > GOV2 > GOV1 > IGV2), size, presence of red spot signs, and Child-Pugh stage [45]. (B2)
- Gastroesophageal and gastric varices may hint at the presence of PVT and/or splanchnic vein thrombosis, which should be investigated. (B2)
- The indications for endoscopic therapy (i.e., cyanoacrylate injection for GOV2 and IGV1; band ligation or cyanoacrylate injection for GOV1; IGV 2 are rare, and treatment should be individualized) are described in **Chap. 5, 6 and 7**.

### Portal-hypertensive gastropathy

- Portal hypertensive gastropathy (PHG) is defined as a macroscopically visible mosaic/cobblestone-like pattern of the gastric mucosa (usually fundus or corpus) [45] (B1) and correlates with the Child-Pugh stage [46]. (B2)
- PHG should be differentiated into mild and severe (i.e., red marks or active bleeding) PHG [45]. (B2)
- Besides the use of vasoactive treatment (see **Chap. 6**) (B1), endoscopic argon plasma coagulation (APC) or haemostatic powder (e.g., Hemospray and Nexpowder) may be applied to treat acute bleeding from PHG [46, 47]. (C1)
- In patients with chronic bleeding, NSBB therapy and iron supplementation should be administered [45]. (B1)
- TIPS and liver transplantation are effective second-line therapies [45]. (B1)

### Gastric antral vascular ectasia

- Gastric antral vascular ectasia (GAVE) is a distinct entity that is endoscopically characterized by columns of erythematous (mild) or hemorrhagic (severe) lesions in a ‘watermelon’ or diffuse pattern (in the latter case, histology may help to confirm the diagnosis) [45]. (B1)
- GAVE may be flat, elevated, or even nodular.
- Notably, GAVE commonly occurs in patients without ACLD/portal hypertension [45] and therapies aiming at ameliorating portal hypertension are ineffective. (B1)
- APC, radiofrequency ablation and—in particular for nodular GAVE—banding [48] may decrease blood loss [45]. (B1)

## 5. Prevention of first hepatic decompensation and portal hypertensive bleeding

- Non-selective betablockers (NSBB)s are **not** indicated for preventing complications of portal hypertension in cACLD patients without CSPH, as both the risk of events [49] and the magnitude of the HVPG-lowering effect of NSBB therapy [50] are negligible in the absence of CSPH. (B1)
- Compensated patients with CSPH or esophageal/gastroesophageal/gastric varices [30]—the latter indicate the presence of CSPH—should be treated with NSBBs to prevent first hepatic decompensation (notably, most commonly ascites) [2]. (B1)
- Due its higher efficacy in lowering HVPG [51], carvedilol (6.25 mg q.d., titrated to 6.25 mg b.i.d or 12.5 mg q.d.) is the NSBB of choice for cACLD. Propranolol should be reserved for those who are intolerants to carvedilol (i.e., systolic blood pressure < 90 mm Hg on carvedilol and/or symptomatic hypotension) [2]. (B1)
- In general, patients on NSBB therapy are **not** required to undergo EGD, as the absence/presence of varices has no therapeutic consequences [2]. However, EGD may be performed due to upper GI symptoms and/or local preferences. (B2)
- Patients with contraindications for or intolerance to NSBB therapy should undergo a screening EGD and endoscopic surveillance, as per Billroth III [45] (B1):
  - No varices on index endoscopy: Repeat after 2 and 3 years in patients with and without an ongoing (i.e., unresolved) primary aetiological factor/cofactor, respectively.
  - Small varices on index endoscopy: Repeat after 1 and 2 years in patients with and without an ongoing (i.e., unresolved) primary aetiological factor/cofactor, respectively.
- Patients with contraindications for or intolerance to NSBB therapy and large esophageal/GOV1 should undergo endoscopic band ligation [2]. (A1)
- In patients with contraindications for or intolerance to NSBB therapy and GOV2/IGV1, endoscopic cyanoacrylate injection should be discussed on a case-by-case basis and performed in experienced centers. (B1)
- Notably, in those without a history of variceal bleeding, endoscopic therapies for its prevention are only indicated in case of contraindications for or intolerance to NSBB therapy (or, where applicable, hemodynamic non-response to NSBBs), as they do **not** prevent non-bleeding first decompensation [2]. (B1)

## 6. Acute variceal bleeding

**Fig. 2:**
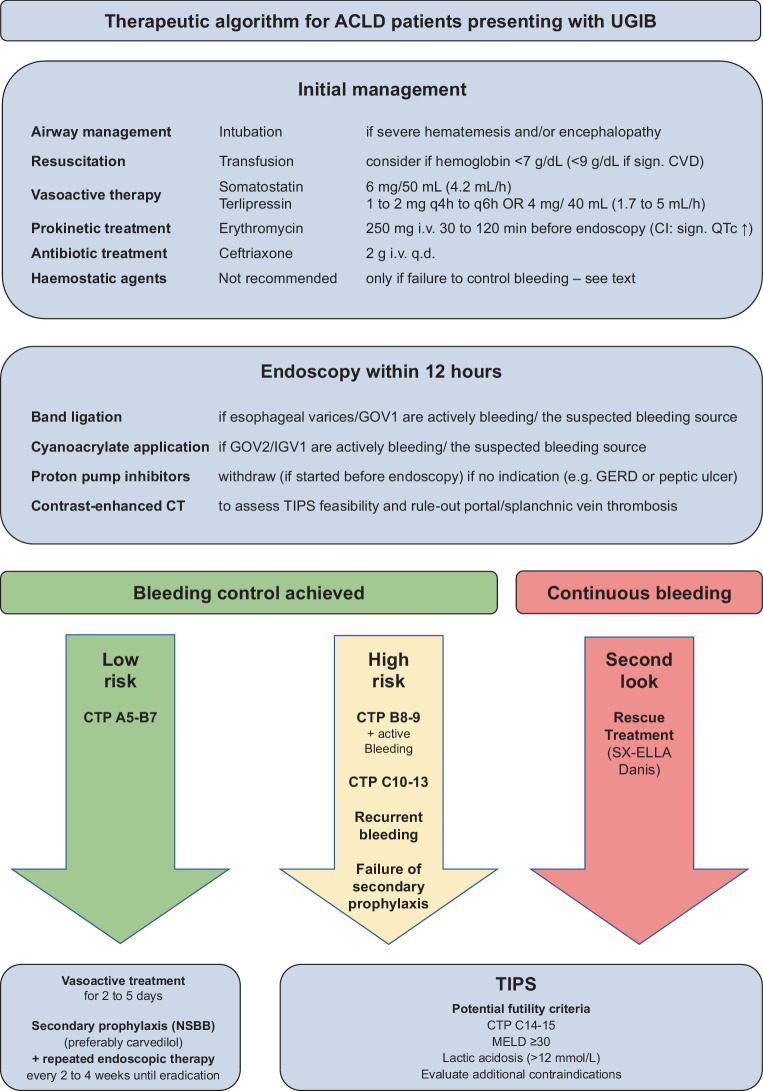
Therapeutic algorithm for the management of advanced chronic liver disease (*ACLD*) patients presenting with upper gastrointestinal bleeding (*UGIB*)

Acute variceal bleeding (AVB) is a severe complication of portal hypertension and its 6‑week mortality has been reported as 15–20% [52]; prognosis is primarily dependent on hepatic function (i.e., MELD [52] and Child-Pugh, with the latter guiding risk stratification/preemptive TIPS placement [53]). As summary of the following recommendations is given in Fig. 2.

### Pre-endoscopy management

- Hemodynamic stabilization, airway management, and medical therapy have priority over endoscopy in patients presenting with a suspicion of AVB [45]. (A1)
- A restrictive transfusion policy aiming for a hemoglobin of 7–8 g/dL is suggested in hemodynamically stable patients without cardiovascular disease (CVD) [54]. Thus, the threshold for red blood cell transfusion is usually 7 g/dL for those without symptomatic CVD (A1), while it may be increased to 9 g/dL in those with CVD. (B1)
- Variceal bleeding is due to portal hypertension and treatment should be focused on lowering portal pressure rather than correcting coagulation abnormalities. Routine coagulation tests do **not** accurately reflect hemostasis. In general, substitution of fresh frozen plasma, recombinant VIIa, or tranexamic acid are **not** recommended. Moreover, in the absence of failure to control bleeding, there is **no** indication to correct abnormalities in coagulation tests by platelet transfusion, prothrombin complex concentrates, or fibrinogen; in those with failure to control bleeding decisions should be made on a case-by-case basis [55]. (B1)
- Intubation is recommended before endoscopy in patients with altered consciousness and those actively vomiting blood [2]. (C1)
- In suspected variceal bleeding, vasoactive drugs, i.e, terlipressin (1–2 mg every 4–6 h), somatostatin (6 mg/50 mL; continuous infusion with 4.2 mL/h), or octreotide, are equally effective [56] and should be started as soon as possible and continued until a portal hypertension-related bleeding source has been endoscopically excluded, or if confirmed, for 2–5 days (A1) [45].
- The pharmacological properties of terlipressin support its continuous infusion; although clinical evidence is limited [57, 58], a dose of 1.7 mL/hour of 4 mg/40 mL (or higher—up to 5 mL/hour) may be suitable to control bleeding. (C1)
- Antibiotic prophylaxis, e.g., i.v. ceftriaxone 1 g q.d. (A1), which may be increased to therapeutic doses of 2–4 g q.d. in clinical practice (D2), is an integral part of therapy for patients with cirrhosis presenting with upper gastrointestinal bleeding and should be instituted from admission [45].
- In the absence of contraindications (significant QTc prolongation), pre-endoscopy infusion of erythromycin (i.v. 250 mg 30–120 min before endoscopy) should be considered [45]. (B1)
- Proton pump inhibitors (PPI), when started before endoscopy, should be stopped when portal hypertension-related bleeding has been confirmed, unless there is an evidence-based indication to continue treatment [2]. (C1)

### Endoscopy

- Following hemodynamic resuscitation and, if required, intubation, patients with suspected AVB should undergo upper endoscopy within 12 h of presentation. If the patient is haemodynamically unstable, endoscopy should be performed as soon as safely possible [2]. (C1)
- The availability of an on-call GI endoscopist proficient in endoscopic hemostasis and on-call support staff with technical expertise in the usage of endoscopic devices, enabling performance of endoscopy on a 24/7 basis, is recommended. Trainees performing the procedure must always be supervised by a GI endoscopist [2]. (C1)
- Active bleeding at endoscopy (defined as blood emanating from a varix, despite vasoactive therapy) is predictive of failure to control bleeding and may have therapeutic implications (e.g., pre-emptive TIPS placement), and thus, should always be noted on the endoscopy report [2]. (B1)
- The endoscopy report should include the information on the applied vasoactive treatment [2]. (B1)
- EVL is the recommended form of endoscopic therapy for acute esophageal variceal bleeding [45]. (A1)
- EVL or cyanoacrylate injection are recommended for acute bleeding from GOV1 [45]. (C1)
- Endoscopic cyanoacrylate injection is recommended for acute bleeding from GOV2 and IGV1 [45]. (C1)
- IGV2 are rare and treatment should be individualized. (C1)
- Based on current evidence, haemostatic powder is **not** recommended as a first-line endoscopic therapy for AVB [2]. (B1)
- In refractory bleeding from esophageal varices, self-expandable metal stents (SEMS) should be used. If not available or applicable, balloon tamponade (Sengstaken-Blakemore tube) should be used as last resort, while Linton-Nachlas tube should be applied for fundal varices. Balloon tamponade must only be performed in an intubated patient to avoid aspiration. For esophageal varices, SEMS are as efficacious but safer than balloon tamponade and allow a longer dwell time (up to 7 days for SX-ELLA Danis) [59]. Notably, these are bridging therapies to definite treatment, i.e., rescue TIPS placement [60]. (B1)

### Post-endoscopy management

- Patients with AVB should be managed in intensive or intermediate care units [2]. (C1)
- Vasoactive drugs should be continued for 2–5 days [45]. (B1)
- Lactulose should be administered to facilitate the removal of blood from the digestive tract to prevent/treat hepatic encephalopathy [2]. (B1)
- All patients with AVB should undergo abdominal imaging, preferably contrast-enhanced cross-sectional imaging (CT or MRI) to exclude PVT/splanchnic vein thrombosis and hepatocellular carcinoma as well as to map portosystemic collaterals to guide treatment [2]. (C1)
- For recommendations regarding pre-emptive (in high-risk patients, if bleeding is controlled by vasoactive treatment and/or endoscopy) and rescue (in case of failure to control bleeding) TIPS, please see the **Chap. 11**.

## 7. Prevention of further decompensation

Disease progression in patients with decompensated cirrhosis comprises new onset of specific complications or extrahepatic organ dysfunction/failure. The former is known as ‘further decompensation’ and the latter as ‘acute-on-chronic liver failure’ (ACLF). Both conditions have a negative impact on prognosis, require specific management, and should prompt evaluation of the patient for an etiological therapy and for liver transplantation candidacy.

### Definition of ‘further decompensation’

- Further decompensation in cirrhosis represents an advanced prognostic stage defined by any of the following: (B1)
  - Development of a second portal hypertension-driven decompensating event (ascites, AVB, or hepatic encephalopathy [HE]) and/or jaundice (bilirubin ≥ 5 mg/dL).
  - Development of recurrent variceal bleeding, recurrent ascites (requirement of ≥ 3 large-volume paracenteses within 1 year), recurrent HE, spontaneous bacterial peritonitis (SBP) and/or hepatorenal syndrome-acute kidney injury (HRS-AKI).
  - In patients presenting with AVB alone, if ascites, HE, or jaundice develop *after* recovery from the bleeding episode, but not if these events occur *around* the time of bleeding.
- PVT may be associated with further decompensation and should thus, be actively screened for, but does not define further decompensation.

### Definition of acute-on-chronic liver failure (ACLF)

Several definitions of acute-on-chronic liver failure (ACLF) have been proposed by societies from different regions of the world. The European Association for the Study of the Liver (EASL) has endorsed [61] the definitions proposed by the European Foundation for the study of Chronic Liver Failure (EF-CLIF) [62], which requires the presence of cirrhosis. According to the EF-CLIF definition, the development of organ dysfunctions/failures discriminates ACLF from acute decompensation (AD), i.e., hospitalization for first/further hepatic decompensation.

This is in contrast to the definition of the Asian Pacific Association for the Study of the Liver (APASL) [63], for which any pre-existing chronic liver disease suffices to define subsequent ACLF, if an acute hepatic insult manifesting as jaundice (bilirubin ≥ 5 mg/dL) and coagulopathy (INR ≥ 1.5 or prothrombin activity < 40%) complicated within 4 weeks by ascites grade ≥ 2 and/or overt HE.

The Billroth IV consensus panel endorses the EF-CLIF definition of ACLF [62]:

- ACLF defines a condition occurring in patients with cirrhosis in response to a hepatic or extrahepatic insult causing liver failure and/or extrahepatic organ failure [61]. (B1)
- ACLF is a life-threatening condition associated with high short term (28-day) mortality [61]. (B1)
- ACLF is commonly triggered by severe alcoholic hepatitis or infections, however, the precipitating event may also be unknown. (B1)
- Hepatic and extrahepatic organ dysfunction/failure should be defined by EF-CLIF criteria, as shown in Table 2 [61]. (B1)
- ACLF is a highly dynamic condition that may fully recover, but also deteriorate to irreversible multiorgan failure and death, and thus, close and at least daily monitoring of liver and extrahepatic organ function is required [61]. (B1)
- As of 04/2023, there is no specific treatment approved for ACLF [61]. (B1)
- Patients with ACLF should be considered for ICU management [61]. (C1)
- Patients with ACLF may be candidates for liver transplantation and patients should be presented to a transplant center [61]. (C1)
- Rapidly deteriorating ACLF and ACLF-3b—in particular if persistent—may indicate therapeutic futility [61]. (B1)

**Table 2:** Diagnostic criteria for ACLF and grading

Organ system	Variable	Dysfunction	Failure
Liver	Bilirubin (mg/dL)	≥ 6.0 to < 12	**≥** **12**
Kidney	Creatinine (mg/dL)	*>* *1.5 to <* *2.0*	**≥** **2.0** **or use of RRT**
Brain	HE West Haven Grade	*I–II*	**III–IV** **or intubation for HE**
Coagulation	INR	≥ 2.0 to < 2.5	**≥** **2.5**
Circulation	MAP (mm Hg)	< 70	**Use of Vasopressors** **(not considering Terlipressin)**
Lung	PaO2/FiO2 SpO2/FiO2	201 to 300 215 to 357	**≤** **200** **≤** **214** **or mechanical ventilation**
**ACLF grading**			
–	Grade 1	1a—Single renal failure (sCre ≥ 2.0 mg/dL) 1b—Isolated liver or coagulation failure combined with either (i) renal dysfunction (sCre 1.5 to < 2.0 mg/dL) or (ii) brain dysfunction (HE I–II)	
	Grade 2	2 organ failures	
	Grade 3	3a—3 organ failures 3b—≥ 4 organ failures	

### Definition of cirrhosis recompensation

The concept of recompensation implies that there is at least partial regression of the structural and functional changes of cirrhosis after removal/suppression of the primary aetiological factor [2]. (B1)

- The definition of recompensation is based on Baveno VII [2] expert consensus and requires fulfilment of all the following criteria: (C2)
  - Removal/suppression of the primary aetiological factor (i.e., HCV-cure, HBV-suppression in the absence of HDV infection, and abstinence from alcohol).
  - Resolution of ascites (off diuretics), HE (off lactulose/rifaximin/L-ornithine L‑aspartate (LOLA)), and/or 12-months without recurrent AVB (carvedilol or conventional NSBBs are not required to have been withdrawn);
  - Stable improvement of liver function tests (bilirubin, INR, and albumin).
- The criteria for recompensation in patients with cirrhosis due to other aetiologies are yet to be established. (D1)
- Resolution of clinical complications after TIPS *per se* does not confer recompensation. (C2)
- Because CSPH may persist despite recompensation, NSBBs should not be discontinued unless CSPH has resolved. (B1)

### Preventing recurrent variceal haemorrhage (secondary prophylaxis)

- First-line therapy for the prevention of recurrent AVB is the combination of carvedilol (alternatively conventional NSBBs) plus EVL [2, 64]. (B1)
- TIPS is the treatment of choice in patients who rebleed despite sufficient secondary prophylaxis using carvedilol (or conventional NSBBs) plus EVL [2]. (B1)
- In patients who cannot get/tolerate EVL or carvedilol (or conventional NSBBs), any of these therapies can be maintained alone (B1), but TIPS should be considered in patients with recurrent ascites [2]. (A1)
- In patients who bleed despite adherence to carvedilol (or conventional NSBBs), the combination of carvedilol (or conventional NSBBs) and EVL is recommended (B1), but TIPS should be considered in those with recurrent ascites [2]. (A1)
- In patients with haemodynamic non-response (HVPG-decrease < 20% from baseline [27]) to carvedilol and very high HVPG (i.e., ≥ 20 mm Hg) TIPS may be considered for secondary prophylaxis on a case-by-case basis [65, 66]. (D2)

### Definition* and diagnosis of hepatic encephalopathy (HE)*

- Hepatic encephalopathy (HE) can occur in patients with acute liver failure (type A), due to presence of portosystemic shunts (type B), and due to cirrhosis with hepatic dysfunction (type C) [67]. (B1)
- Covert HE can only be identified by neurophysiological or neuropsychological testing [67]. Notably, local cut-offs are required for neuropsychological tests (e.g., < 20 different animals within 1 min for the simplified animal naming test [68], which can be performed as a bedside test). (B1)
- Recurrent HE is defined as ≥ 2 HE bouts within 6 months, while HE that occurs less frequently is considered as episodic HE [67]. (B1)
- Persistent HE is defined if a patient does not return to baseline performance between bouts [67]. (B1)
- The severity of overt HE should be graded according to the West Haven criteria as II–IV [67]. (B1)
- Normal plasma ammonia levels usually rule-out overt HE [67]. (B1)
- CT and MRI should be performed in unclear cases or if other cerebral conditions are suspected (B1). Cerebral imaging is not diagnostic of HE, but rules-out differential diagnoses and may reveal cerebral oedema [67]. (C1)

### Treatment of first, recurrent, and persistent HE

- Covert (B1) and overt HE should be treated with lactulose (titrated to achieve 2–3 bowel movements/d) [67]. (A1)
- Precipitating factors of HE should be identified and treated (B1), most importantly dehydration (e.g., by diuretic overuse), infections, and acute gastrointestinal bleeding [67].
- In patients with HE, vitamin and micro-/macro-nutrient deficiencies should be identified and treated [67]. (C1)
- HE should *not* prompt a reduction of protein intake or enteral nutrition. (C1)
- Patients with HE West Haven Grade III–IV are at risk for aspiration and ICU management/intubation must be considered [67]. (B1)
- Lactulose is recommended as secondary prophylaxis of HE [67]. (A1)
- Rifaximin can be added to lactulose in case of recurrent or persistent HE. (B1)
- L‑ornithin L‑aspartate (LOLA) can be added to lactulose in case of recurrent or persistent HE. (C1)
- Patients with recurrent or persistent HE should be evaluated for liver transplantation. (B1)
- Embolization/occlusion of large portosystemic shunts/collaterals should be considered in patients with recurrent or persistent HE. (C1) After closure of shunts, endoscopy should be performed to screen for varices, if the patient is not on carvedilol or conventional NSBBs for primary bleeding prophylaxis. (D1)
- After an episode of overt HE, patients should be provided with information on the risks associated with driving. (D1)

### Preventing further decompensation in patients with ascites or HE

- Decompensated patients with ascites or HE who are not on carvedilol (or conventional NSBBs) should undergo screening endoscopy. (B1)
- In decompensated patients with ascites or HE and low-risk varices (small [< 5 mm], no red signs, not Child-Pugh C), carvedilol (or conventional NSBBs) may be used to prevent first variceal haemorrhage. (B1)
- In decompensated patients with ascites or HE and high-risk varices (large varices [≥ 5 mm], or red spot signs, or Child-Pugh C), prevention of first variceal haemorrhage with carvedilol (or conventional NSBBs) is indicated (B1) and preferred over EVL.

### Role of infections in decompensated cirrhosis

- Bacterial infections are common in patients with decompensated cirrhosis and may cause further decompensation [2, 61]. (B1)
- In all patients hospitalised with AD, bacterial infections should be ruled-out. The minimal work-up for infections should include diagnostic paracentesis, cultures of ascites, blood, and urine, chest X‑ray, and skin examination. Nosocomial infections are defined by an onset 72 h after hospitalization [2, 61]. (B1)
- Patients with bacterial infections should be promptly treated with antibiotics. If no response to antibiotics is observed, consider fungal and viral infections [2, 61]. (C1)
- A guidance for empirical antibiotic treatment for community-acquired and nosocomial bacterial infections is given in Table 3. Empirical antibiotic therapy should be started immediately and consider the local antimicrobial resistance profile, the clinical context (i.e., community-acquired vs. nosocomial), and the severity of infection (i.e., presence of septic shock) [2, 61, 69, 70]. (B1)
- In case of unclear bacterial infections, an empirical antibiotic treatment strategy similar as for SBP should be considered. (C1)

**Table 3:** Guidance for empirical antibiotic therapy for non-SBP infections in cirrhosis

Type of infection	Community-acquired infections	Nosocomial infections ^a^
*Cellulitis*	‘Erysipel’: Penicillin G (i.v.)/V (p.o.) ‘Phlegmone’: Cefazolin (i.v.)/cefalexin (p.o.), flucloxacillin	
*Urinary tract infections*	Uncomplicated: Pivmecillinam, Fosfomycin, ciprofloxacin, or cotrimoxazole	
	If sepsis: Aminopenicillin/beta-lactamase inhibitor or cefotaxime or ceftriaxone	If sepsis: Piperacillin/tazobactam or meropenem ± glycopeptide^b^
*Pneumonia*	Aminopenicillin/beta-lactamase inhibitor or cefotaxime or ceftriaxone ± macrolide or levofloxacin or moxifloxacin	Piperacillin/tazobactam or cefepime or meropenem ± ciprofloxacin/levofloxacin ± glycopeptide ^b^ should be added in case of high MRSA risk ^c^

Dosages of antibiotics have not been formally and specifically investigated or defined in patients with cirrhosis, however, it is advisable to follow standard recommended dosages adopted to renal function

^a^ Recommended also for health-care associated pneumonia and urinary infections

^b^ Glycopeptides must be replaced by linezolid or daptomycin in areas with high prevalence of vancomycin-resistant enterococci (VRE)

^c^ Ventilator-associated pneumonia (VAP), recent antibiotic therapy, nasal MRSA carriage

### The role of sarcopenia and frailty in further decompensation

- Frailty, malnutrition, and sarcopenia have an impact on survival in patients with decompensated cirrhosis. They should be evaluated with available standardised tools [2]. (B1)
- All patients with decompensated cirrhosis should receive nutrition consultation (e.g., optimal daily energy intake should not be lower than 35 kcal/kg actual body weight (BW)/day in non-obese individuals and protein intake should not be lower than 1.2–1.5 g/kg actual BW/day; late-evening oral nutritional supplementation should be recommended [67]) and be advised regarding the benefits of regular exercise [2]. (B1)
- While sarcopenia improves in some patients after TIPS, preprocedural sarcopenia has also been associated with poor outcomes (e.g., HE, less ascites control) and a higher mortality. Therefore, sarcopenia by itself should not be an indication for TIPS [2]. (B1)
- Patients with cirrhosis-associated sarcopenia should be evaluated early for transplant candidacy because liver transplantation improves sarcopenia in most cases, but sarcopenia may deteriorate with further decompensation and then increase mortality, even in those who finally undergo liver transplantation. The severity and course of sarcopenia should be carefully assessed and addressed prior to liver transplantation [67]. (B1)

## 8. Management of ascites and hepatic hydrothorax

Hepatic decompensation includes development of clinically overt ascites or hepatic hydrothorax related to portal hypertension (as suggested by a serum ascites albumin gradient [SAAG] > 1.1 g/dL). Mortality in patients with cirrhosis developing ascites is 15–20% within 1 year and 44% within 5 years [71, 72]. Treating ascites also improves quality of life and the occurrence of SBP is unlikely in patients without ascites. Important definitions, grading and treatment are summarized in Table 4**.**

**Table 4:** Diagnosis and therapy of ascites

Uncomplicated ascites				Recurrent ascites	Refractory ascites
	Grade 1	Grade 2	Grade 3		
*Definition*	Mild ascites only detectable by ultrasound	Moderate ascites evident by moderate abdominal distension	Tense ascites with marked abdominal distension	Ascites that is associated with frequent LVP (at least 3 within 12 months) despite optimal treatment	Ascites that cannot be mobilized or with early recurrence due to lack of response to sodium restriction and diuretic treatment; impaired urinary sodium excretion (< 80 mmol/24 h); spot urinary sodium/potassium ratio < 2.5
*Treatment*	Moderate sodium restriction	Moderate sodium restriction and MRAs, if not responsive additional loop diuretic	Paracentesis, sodium restriction, and diuretics Evaluation for OLT	TIPS or repetitive large volume paracentesis OLT must be considered	
*Avoid*	NSAIDs, angiotensin converting enzyme inhibitors, angiotensin receptor blockers, α1-adrenergic receptor blockers, aminoglycosides		NSAIDs, angiotensin converting enzyme inhibitors, angiotensin receptor blockers α1-adrenergic receptor blockers, aminoglycosides, carvedilol if hypotensive, propranolol with caution (not more than 80 mg/day)		

### Diagnostic approach in patients with ascites

- Ascites should be graded according to the International Ascites Club guidelines into uncomplicated (grade 1: only visible on ultrasound, grade 2: moderate ascites, grade 3: tense ascites), recurrent (the need for large volume paracentesis (LVP) for ≥ 3 times within a time period of 12 months despite optimal medical therapy), and refractory ascites (ascites that requires repetitive LVP in patients who do not respond or are intolerant to diuretic therapy) [73, 74]. (B1)
- Paracentesis is indicated in patients presenting with (i) ascites for the first time, (ii) grade 3 ascites, (iii) ascites at non-elective hospital admission regardless of the reason, and (iv) ascites with signs of clinical deterioration (such as GI bleeding, shock, fever or other signs of systemic inflammation, abdominal symptoms, hepatic encephalopathy, and in patients with worsening liver or renal function) [74]. (B1)
- Paracentesis is a low-risk procedure that rarely leads to serious bleeding complications; therefore, substitution of coagulation factors or platelets is **not** necessary regardless of laboratory coagulation tests or platelet count [55, 75]. (B2)
- Investigation of ascites should include at least the determination of ascitic neutrophil count, total protein concentration, and the serum-ascites albumin gradient. Uncomplicated ascites due to portal hypertension is expected to show a neutrophil count < 250/µL, a SAAG > 1.1 g/dL [76] and a protein level < 2.5 g/dL. The SAAG is calculated by subtracting the ascitic fluid albumin level from the serum albumin level (determined simultaneously). (B1)
- Additionally, aerobic and anaerobic blood culture bottles should be inoculated with ascitic fluid for microbiological diagnosis of SBP or bacterascites (neutrophil count < 250/µL but positive ascites fluid culture) and to guide subsequent antibiotic treatment. (B1)
- Ascites can develop/aggravate secondary to HCC, PVT, or splanchnic vein thrombosis; therefore, an ultrasound examination should be performed for exclusion in patients with grade 2/3 ascites. (B1)

### Therapy of uncomplicated ascites

- Initial therapy of patients with cirrhosis and ascites consists of moderate sodium restriction and diuretic treatment. (A1)
- Moderate sodium restriction (90 mmol NaCl/day, corresponding to 5.2 g NaCl/day) is usually equivalent to a no added salt diet with avoidance of pre-cooked meals. Extreme sodium restriction to less than 5 g NaCl/day is **not** recommended due to the risks of diuretic-induced hyponatremia, renal failure, and aggravation of malnutrition that is commonly present in these patients [77–79]. (B1)
- Due to the central role of secondary hyperaldosteronism in the development of cirrhosis-associated ascites, mineralocorticoid receptor antagonists (MRAs, especially spironolactone) are considered as first-line therapies. Patients with a first episode of moderate ascites can be treated with spironolactone alone starting at 100 mg/day with stepwise increases every 3–5 days to a maximum dose of 400 mg; especially in the outpatient setting due to less frequent dose adjustments needed [80]. (B2)
- In patients who (i) do not respond to MRAs as defined by a decrease in body weight of less than 2 kg/week, (ii) develop hyperkalaemia or (iii) present with long-standing, recurrent or tense ascites, furosemide should be added or a combination therapy consisting of spironolactone and furosemide should be started [81]. Furosemide should be started with 40 mg/day; a daily cumulative dose of 160 mg furosemide should not be exceeded. (B1)
- Furosemide should not be administered intravenously as a bolus in patients with cirrhosis, because of risk of deterioration in the glomerular filtration rate (GFR) [82]. (B1)
- Rapid weight loss during diuretic therapy might increase the risk of hypovolemia, acute kidney injury (AKI), and HE, and thus, weight loss during diuretic therapy should not exceed 0.5 kg/day in patients without oedema and 1 kg/day in patients with oedema. Patients should be encouraged to monitor body weight daily. (B2)
- Eplerenone is an alternative especially for men with gynaecomastia. 100 mg of spironolactone is considered roughly equivalent to 50 mg of eplerenone [83]. Furthermore, torasemide can be used as an alternative to furosemide, allowing for less frequent dosing [84]. (B2)
- Vaptans are not indicated for the management of portal-hypertensive ascites [85]. (B2)
- After initiation or adaptation of diuretic therapy, renal function and electrolytes should be monitored. (B1)
- After mobilization of ascites, diuretics should gradually be tapered to the lowest doses capable of maintaining BW with minimal or no ascites. Removal/suppression of the primary aetiological factor should be encouraged, if possible, to facilitate control of ascites in these patients. (B2)
- In patients with hypervolemic hyponatremia, fluid restriction and monitoring are recommended when plasma sodium levels fall below 125 mmol/L. Furthermore, diuretics should at least be temporarily withdrawn when serum sodium concentration decreases below 120–125 mmol/L. (C2)
- Substitution with hypertonic NaCl solutions should be avoided since it may promote volume overload and worsen ascites and oedema. It should be limited to severely symptomatic hyponatremia, as defined by life-threatening manifestations, cardio-respiratory distress, somnolence, seizures, and coma. (C2)
- In patients with tense ascites (grade 3), large-volume paracentesis (LVP) is the treatment of choice and should be followed by diuretic therapy. Total paracentesis should be carried out as a single procedure, even when a large volume of ascites is present, if it is hemodynamically tolerated by the patient. (B1)
- Plasma volume expansion using albumin is recommended in all patients undergoing LVP (i.e., if more than 5 L of ascites have been removed) for prevention of circulatory dysfunction [86]. Albumin at a dose of 8–10 g/L ascites removed (i.e., 100 mL 20% albumin per 2.5 L of ascitic fluid) should be administered. Removal of less than 5 L does not appear to have significant hemodynamic consequences [87] (A1), however, in patients with hemodynamic instability (systolic blood pressure < 90 mm Hg), hyponatremia < 130 mmol/L and/or presence of AKI, albumin infusion should be strongly considered for paracentesis < 5 L [88]. (C2)
- The administration of nonsteroidal anti-inflammatory drugs (NSAIDs) in patients with ascites due to portal hypertension can lead to renal failure and should therefore be avoided [89]. The same is true for angiotensin receptor blockers, angiotensin converting enzyme inhibitors and α1-adrenergic blockers besides carvedilol [90]. Aminoglycosides should be avoided whenever possible [91]. (B1)
- In the absence of evidence-based indications, PPI should **not** be used in patients with ascites since PPI might be associated with a higher risk of infection [92]. (B2)
- Ascites is **not** a contraindication for NSBBs, but they should be used with caution, especially carvedilol or high doses of propranolol (> 80 mg/day) in recurrent/refractory ascites [93]. NSBBs should be temporarily dose-reduced or discontinued in case of persistently low blood pressure (systolic blood pressure <90 mm Hg or mean arterial pressure < 65 mm Hg) and in patients who develop an acute intercurrent condition such as bleeding or HRS-AKI [29, 94, 95]. (C2)
- The benefit of long-term albumin administration remains controversial and therefore, no recommendation can be made for its use in routine clinical practice [96, 97]. (C2)

### Recurrent ascites

- TIPS should be considered in patients with recurrent ascites (≥ 3 LVP within 1 year) since it improved transplant-free survival in a small randomized study [98] (B1).

### Refractory ascites

- Refractory ascites is associated with poor survival of only 50% at 6 months [99]. Refractory ascites is defined by the ICA [73] as
  - ascites that cannot be mobilized by intensive diuretic therapy (up to a maximum cumulative dose of 400 mg spironolactone and 160 mg furosemide/day) and confirmed dietary sodium restriction (diuretic-resistant ascites),
  - or as ascites that rapidly reaccumulates after therapeutic paracentesis (within 4 weeks),
  - or as the situation, where the maximum dose of diuretics cannot be administered due to side effects, such as electrolyte imbalance, renal failure, and HE (diuretic-intractable ascites).
- A characteristic feature of refractory ascites is impaired urinary sodium excretion despite maximum tolerated doses of diuretics [100]. Since urine collection for 24 h is cumbersome, a spot urinary sodium/potassium ratio < 2.5 is a reasonable surrogate for diuretic-resistant ascites [101]. Diuretic treatment should be continued only when urinary sodium excretion under diuretic therapy is greater than 30 mmol/day [102]. (B2)
- Due to the poor prognosis of patients with refractory ascites, liver transplantation should be considered. (A1)
- Patients with refractory ascites should be evaluated for TIPS, since TIPS is associated with improved survival [103–106], especially when smaller diameter covered stents are used [107–109]. (B1)
- If TIPS is contraindicated or refused by the patient, repetitive LVP in combination with albumin substitution, sodium restriction, and diuretic therapy should be performed. (B1)
- Alfapump® [110–113], a low-flow pump system to remove ascites from the peritoneal cavity into the bladder, or tunnelled peritoneal drainage systems [113] are **not** expected to improve survival in patients with refractory ascites, and thus, should be limited to non-transplantable patients who are poor candidates for TIPS. (C2)

### Hepatic hydrothorax

- Hepatic hydrothorax represents a (usually right-sided) pleural effusion in patients with decompensated cirrhosis in the absence of any other pleural, pulmonary, or cardiac disease [114]. (B1)
- Diagnostic thoracentesis of hepatic hydrothorax should be performed at first diagnosis and includes similar testing as for ascitic fluid. (B1)
- Spontaneous bacterial pleuritis can be diagnosed if the neutrophil cell count is > 500 or > 250 cells/µl with a positive culture after exclusion of parapneumonic pleural effusion or empyema and should be treated similar to SBP [115]. (B1)
- Hepatic hydrothorax should be primarily treated with salt restriction and diuretics [116]. (A1)
- If patients are presenting with dyspnoea, repeated therapeutic thoracentesis is indicated for symptomatic relief [116]. (A1) However, insertion of chest tubes is **not** recommended due to high complication risk including infection, electrolyte disturbances, and renal dysfunction [117, 118]. (B1)
- TIPS should be considered for recurrent hepatic hydrothorax not responsive to diuretic therapy [119, 120]. (B1)
- Patients with recurrent hepatic hydrothorax should be evaluated for liver transplantation [121]. (A1)
- Pleurodesis, mesh repair of diaphragmatic defects, or insertion of tunnelled pleural drainage systems may be considered in selected patients with recurrent hepatic hydrothorax if TIPS and liver transplantation are not an option [122–124]. (C2)

## 9. Treatment of spontaneous bacterial peritonitis (SBP)

- SBP is bacterial infection of ascitic fluid and defined by a neutrophil count > 250/mm^3^. A positive ascitic fluid culture with a neutrophil count ≤ 250/mm^3^ is termed bacterascites. SBP can be categorized into community-acquired and nosocomial SBP. Nosocomial SBP is defined by an onset 72 h after hospitalization.
- Diagnostic workup for SBP is recommended in patients with cirrhosis and ascites (i) developing it for the first time, (ii) at unscheduled hospital admission, (iii) with signs of systemic infection, (iv) with further decompensation (e.g. variceal bleeding or hepatic encephalopathy), or (v) with deterioration of hepatic or renal function [4]. Delayed diagnosis of SBP is associated with increased mortality, highlighting the importance of timely paracentesis [125]. (B1)
- Screening for SBP comprises sampling of ascitic fluid and blood in aerobic and anaerobic blood culture bottles for adopting antibiotic treatment, if necessary. (B1)
- Antibiotic treatment is recommended in all patients diagnosed with SBP. Patients with bacterascites should receive antibiotic treatment if they present with signs of systemic infection or if bacterascites is confirmed at a second paracentesis. Empirical antibiotic therapy should be started immediately and consider the local antimicrobial resistance profile, the clinical context (i.e., community-acquired vs. nosocomial), and the severity of infection (i.e., presence of septic shock). (A1)
- Third-generation cephalosporins (e.g., i.v. ceftriaxone 2–4 g q.d.) are recommended as first-line antibiotic treatment for community-acquired SBP in countries with low rates of bacterial resistance (e.g., Austria) [4]. (A1)
- Nosocomial SBP is more likely to harbour resistance to antibiotics. Piperacillin/tazobactam should be given in contexts with a low prevalence of multi-drug resistance (MDR), while carbapenems should be used in contexts with high prevalence of ESBL-producing bacteria [4]. Carbapenems should be combined with either glycopeptides, daptomycin, or linezolid in contexts with a high prevalence of gram-positive MDR bacteria or in patients with septic shock [126]. (B1)
- Severe infections with extended drug resistant (XDR) bacteria may require the use of newer antibiotics. (C2)
- Antibiotic treatment should last at least 5–7 days and be refined according to bacterial culture results. (B1)
- Chinolones should not be used to treat SBP in patients who were on norfloxacin prophylaxis [127]. (B1)
- To prevent the development of HRS-AKI, 1.5 g/kg BW albumin should be administered in patients with SBP at the time of diagnosis, plus 1 g/kg on day three [128]. (A1).
- Blood pressure should be carefully monitored in patients with SBP. NSBBs should be discontinued in case of systolic blood pressure < 90 mm Hg, mean arterial pressure < 65 mm Hg, or HRS-AKI [129, 130]. NSBB should be re-initiated when SBP (± HRS-AKI) and/or arterial hypotension have/has resolved. (B2)
- A second paracentesis should be performed 48 h after initiation of the antibiotic therapy to monitor dynamics of the ascitic fluid neutrophil count [131]. A reduction of ascitic fluid neutrophil count < 25% or worsening clinical symptoms/inflammation markers indicate treatment failure and should trigger consideration of adopting the antibiotic treatment regimen to cover gaps in the antimicrobial spectrum of the initial therapy, as well as relevant MDR. Moreover, fungal infection should be considered in case of non-response to initial antibiotic therapy, in particular in those with septic shock or Child-Pugh C [132]. (B1)
- The use of primary antibiotic prophylaxis should be individualized; norfloxacin 400 mg orally q.d. may be considered in patients with a low ascitic fluid protein concentration (< 15 g/L) and either Child-Pugh ≥ B9 plus serum bilirubin ≥ 3 mg/dL, or an impaired kidney function (serum creatinine [sCre] ≥ 1.2 mg/dL, blood urea nitrogen [BUN] ≥ 25 mg/dL, or serum sodium < 130 mmol/L) [133, 134]. (A2) Lack of evidence and the risk of antibiotic resistance preclude a recommendation of primary antibiotic prophylaxis for patients not fulfilling these criteria.
- The administration of prophylactic norfloxacin (400 mg orally q.d.) is recommended in patients who recovered from an SBP episode [135]. (A1) However, chinolone-based prophylaxis appears to be less efficient in patients colonized with MDR organisms [136].
- In patients who resolve ascites, antibiotic prophylaxis may be discontinued. (C1)
- Based on the currently available evidence, rifaximin cannot be recommended as an alternative to norfloxacin for secondary prophylaxis of SBP [137–141]. (C1)
- In the absence of evidence-based indications, PPI should **not** be used in patients with ascites and a history of SBP [142, 143]. (B2)

## 10. Renal impairment

### Definition, diagnosis and staging of acute kidney injury in ACLD

Acute kidney injury (AKI) is defined as an acute and clinically relevant reduction in the glomerular filtration rate (GFR) [144, 145]. Various causes may result in AKI in patients with ACLD. Among them, prerenal AKI due to hypovolemia (e.g., caused by diuretic overuse, LVP without albumin replacement, or gastrointestinal blood loss) as well as HRS and acute tubular necrosis (ATN) are most common. Pathophysiologically, HRS-AKI results from compromised renal perfusion caused by systemic vasodilatation in patients with ascites and is often aggravated by infections and systemic inflammation [146, 147]. AKI induced by acute tubular necrosis (ATN-AKI) is primarily caused by shock [148] and/or cholemic nephropathy (also known as bile cast nephropathy) [149–152]. Finally, less common causes of AKI such as glomerulonephritis and postrenal obstruction should be considered as differential diagnoses [148]. Since prerenal AKI cases can be successfully treated by plasma volume expansion and postrenal AKI is rare, the main challenge is the differentiation between HRS-AKI and ATN-AKI [153] which may also co-exist.

Diagnosis and staging of AKI in patients with ACLD [154] (B1):

- AKI stage 1: Increase in sCre ≥ 0.3 mg/dL within 48 h to ≥ 1.5- to 2‑fold of the baseline value (obtained as close as possible to the event, up to 3 months in the past)
  - AKI stage 1A: sCre at diagnosis < 1.5 mg/dL
  - AKI stage 1B: sCre at diagnosis ≥ 1.5 mg/dL
- AKI stage 2: sCre > 2- to 3‑fold of the baseline
- AKI stage 3: sCre > 3-fold of the baseline or to ≥ 4 mg/dL with an acute increase ≥ 0.3 mg/dL or need for renal replacement therapy (RRT)
- If no previous sCre value is available, the sCre on admission should be used. In case of impairment of renal function (sCre ≥ 1.5 mg/dL) at time of admission and a clearly identifiable precipitating event, it is reasonable to assume a previously normal renal function, and thus, AKI based on clinical judgement [154].
- In patients with a urinary catheter, an output ≤ 0.5 mL/kg body weight ≥ 6 h may be used to diagnose AKI given its prognostic implications [153, 155, 156].

### Definition and diagnosis of HRS-AKI

**Fig. 3:**
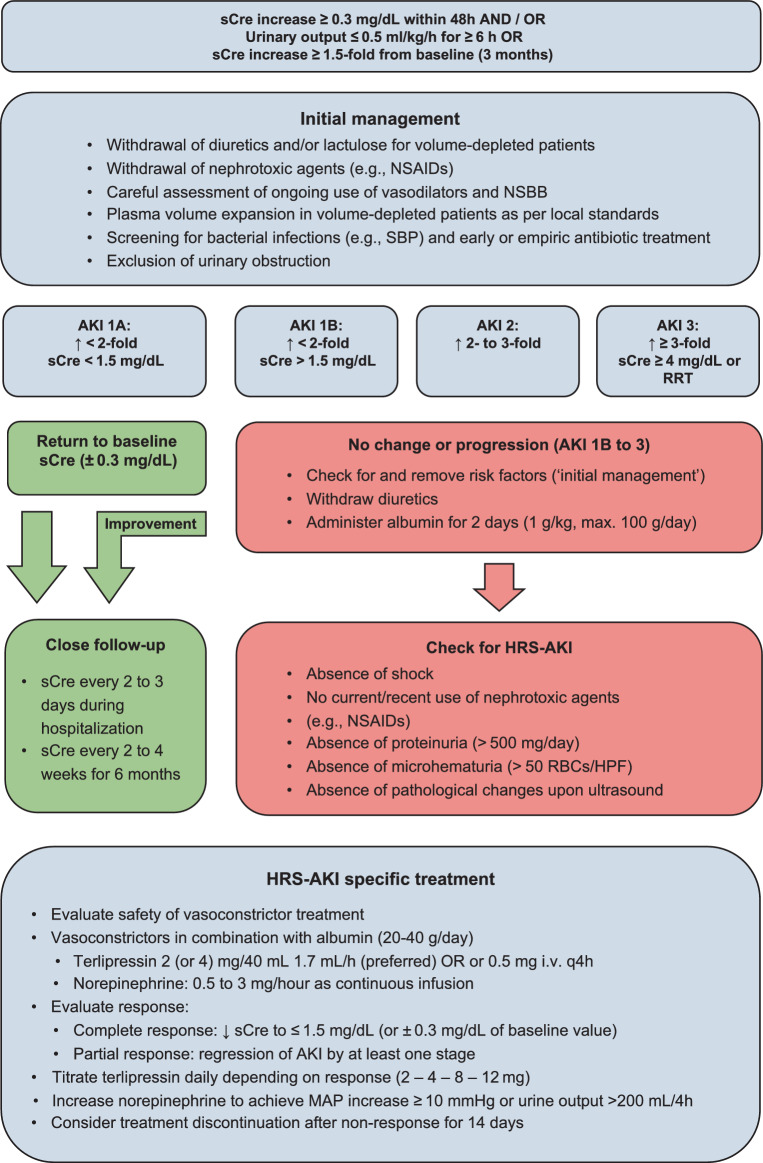
Diagnosis, staging, and management of AKI and in patients with cirrhosis and ascites

- HRS-AKI, previously termed HRS type 1, must be considered in ACLD patients with ascites. As HRS-AKI is a diagnosis of exclusion, other potential (intrinsic) causes of AKI must be ruled-out. Figure 3 demonstrates the diagnostic pathway towards HRS-AKI diagnosis [153] (C1):
  - AKI 1B, as described previously.
  - No improvement in sCre after 2 consecutive days of withdrawal of diuretics and plasma volume expansion with albumin (1 g/kg BW, max. cumulative dose 100 g/day)
  - Absence of shock
  - No current or recent use of nephrotoxic agents (e.g., NSAIDs, aminoglycosides, or contrast media)
  - Exclusion of parenchymal kidney disease, i.e., absence of proteinuria (> 500 mg/day), microhematuria (> 50 RBCs per high power field), and pathological changes upon renal ultrasonography
- HRS-non-AKI (HRS-NAKI, previously HRS type 2) is defined by (non-acute) declines in estimated GFR (eGFR) levels to < 60 mL/min per 1.73 m2. It can be further subclassified depending on the natural history of (non-acute) kidney dysfunction in cirrhosis into an acute kidney disease (HRS-AKD) and a chronic kidney disease (HRS-CKD) phenotype [153]:
  - HRS-AKD: eGFR < 60 mL/min per 1.73 m^2^ for less than three months with a percent increase in sCre < 50% within the last (up to) 3 months
  - HRS-CKD: eGFR < 60 mL/min per 1.73 m^2^ ≥ 3 months

### Management of AKI in ACLD

The initial management should focus on (i) the identification and (ii) the correction of precipitating factors fueling the hemodynamic disturbances in ACLD [154, 157, 158].

- Measures in AKI stage 1A [154, 157] (C1):
  - Review of the entire medication (including over the counter drugs and herbals)
  - Withdrawal of diuretics and reduction or withdrawal of lactulose in case of volume-depletion
  - Withdrawal of potentially nephrotoxic agents (e.g., NSAIDs)
  - Careful assessment of ongoing use or withdrawal of vasodilators and NSBBs [94, 129]
  - Plasma volume expansion in patients with clinically suspected hypovolemia as per local standard
  - Blood transfusion in case AKI origins from gastrointestinal blood loss
  - Extensive search for bacterial infections (e.g., paracentesis to diagnose SBP) with the aim of early antibiotic treatment, if indicated [159]
  - Exclusion of urinary obstruction via ultrasound
- In case of response (return of sCre within 0.3 mg/dL of the baseline value), patients should be followed closely for early identification of potential new episodes of AKI [154, 160] (D1):
  - Close assessment (e.g., every 2 days) of sCre during hospitalization
  - Assessment of sCre every 2–4 weeks during the first 6 months after discharge
- In case of AKI stage 1B, 2 or 3 or progression of stage 1A to a higher stage, patients need to be assessed for the presence of HRS-AKI [154] (B1):
  - Plasma volume expansion with albumin for two consecutive days (1 g/kg BW, max. cumulative dose 100 g/day)

### Management of HRS-AKI and HRS-NAKI

- Patients should be monitored closely (see also **Chap. 7** on the management of ACLF) [148].
- Patients with HRS-AKI stages 1B, 2 and 3 with no complete response within 48 h despite general therapeutic measures and plasma expansion as described above who are considered to have HRS-AKI should be treated with vasoconstrictors in combination with albumin (20–40 g/day). Complete response is defined by a decrease in sCre to a value < 1.5 mg/dL or return to within 0.3 mg/dL of the baseline value [148, 154]. (B1)
- In hypotensive patients (i.e., systolic blood pressure < 90 mm Hg or mean arterial pressure < 65 mm Hg), terlipressin treatment may be initiated before the end of the 48 h period. (D2)
- AKI stage 1A (sCre < 1.5 mg/dL) fulfilling the other diagnostic criteria of HRS-AKI can be treated the same way on a case-by-case basis [154]. (D2)
- Treatment with albumin and terlipressin may also be considered in HRS-NAKI patients who are potential transplant candidates, however, recurrence is common and there is no clear evidence for beneficial effects on pre- and post-transplant outcomes [153, 161–163]. (B2)

### Vasoconstrictor treatment

- Should preferably be administered on IMCU/ICUs. (B1)
- Should preferably be administered via a central venous line under continuous blood pressure and electrocardiography (ECG) monitoring. (B1)
- Non-availability of an IMCU/ICU should, however, **not** defer the timely use of vasoconstrictors in patients with HRS-AKI. (B1)
- Terlipressin is the most extensively studied vasoconstrictor for the treatment of HRS-AKI and therefore recommended [2] (B1).
- A bolus of terlipressin induces a statistically significant reduction in portal pressure over a 3–4-hour period while increasing mean arterial pressure and therefore renal perfusion pressure [164], which translates into an improvement in renal function. (B1)
- The randomized CONFIRM trial found an increased rate of respiratory adverse events under terlipressin (bolus administration) [165]. Consequently, a baseline assessment including a physical exam, an evaluation of fluid status, vital sign assessment with pulse oximetry, a chest X‑ray, and if indicated, a transthoracic echocardiogram (TTE) should be performed in order to minimalize the risk for such events. (B1)
- Careful risk/benefit evaluation prior to treatment with terlipressin and albumin should be performed in patients with ACLF grade 3, pulse oximetry < 90% at room air, or pulmonary oedema on chest X‑ray as these patients may be at highest risk for developing respiratory adverse events. (D2)
- Terlipressin should be used with caution in patients with cardiovascular disease since it may induce ischemia. (B1)
- Surveillance for side effects related to vasoconstriction (ischemia of fingers or skin, abdominal pain, and angina pectoris) should be performed. (B1)
- Patients should also regularly be screened for the development of pulmonary oedema. In case of worsening hypoxia, interrupting or discontinuing terlipressin should be considered. (B1)
- Patients should be monitored for the development of (severe) diarrhoea and hyponatremia. The latter occurs more commonly in patients with less advanced liver disease and (near‑) normal baseline serum sodium levels [166]. In case of significant adverse effects, dose reduction, interruption, or discontinuing of terlipressin should be considered. (B1)
- Compared to bolus administration (initial dose 0.5 mg every 4 h; maximum dose 2 mg every four hours), continuous infusion (initial dose 2 mg/day, e.g., 2 mg/40 mL at 1.7 mL/hour; increased every 48 h according to response; maximum dose 12 mg/day, e.g., 4 mg/40 mL at 5 mL/hour) decreases the rate of (ischaemic) AEs, the mean effective terlipressin dose [167], and, thus, might also decrease costs. Considering the pharmacodynamic profile of terlipressin described above, continuous infusion should be preferred over bolus administration. (A1)
- Terlipressin is particularly beneficial in patients with systemic inflammatory response or sepsis and might also prevent variceal bleeding during the period of discontinuation of NSBBs [168, 169]. (B1)
- Although terlipressin has been consistently shown to improve renal function, its impact on survival is less clear [165]. (A1)
- In the absence of ACLF, norepinephrine (initial dose 0.5 mg/hour; max. dose studied in RCTs 3 mg/hour) may be a comparably effective as compared to terlipressin. A meta-analysis of four RCTs demonstrated similar efficacy in terms of HRS reversal, when compared to terlipressin [170]. (B1)
- In HRS-AKI patients with ACLF, however, terlipressin is superior to norepinephrine and should be preferred [171]. (B1)

### Assessment of response to vasopressor treatment and further management

- Complete response is defined by a decrease in sCre to a value < 1.5 mg/dL or return to within 0.3 mg/dL of the baseline value, while a regression of at least one AKI stage is considered as partial response [154]. (B1)
- Response to terlipressin treatment should be assessed every 2 days. In case of non-response, dose should be increased in a stepwise manner (e.g., 2—4—8—12 mg/day) to a maximum dose of 12 mg/day (continuous infusion: 4 mg/40 mL at 5 mL/hour; bolus administration: 2 mg every 4 h); a more rapid titration may be decided on a case-by-case basis [167, 171] (B1)
- Haemodynamic response to norepinephrine defined as an increase in mean arterial pressure (MAP) of ≥ 10 mm Hg or increase in 4‑hour urine output > 200 ml should be assessed every four hours, if possible. In case of non-response to norepinephrine, dose should be increased by 0.5 mg/hour every 4 h to a maximum dose of 3 mg/hour [172]. (B1)
- In case of complete response, vasoconstrictor treatment should be maintained for 24 h and may be stopped afterwards (B1).
- In patients whose sCre remains at or above the pre-treatment level (non-responders) for 14 days, treatment discontinuation may be considered. (B2)
- In responders, longer treatment durations may be used to prevent early recurrence of HRS-AKI or as a bridging therapy prior to liver transplantation. (D1)
- Recurrent HRS-AKI should be treated in the same way [102]. (D1)

### Role of TIPS in the treatment of HRS-AKI and HRS-NAKI

- TIPS may improve kidney function in patients with HRS-AKI and HRS-NAKI [173–175]. In addition, a relevant proportion of patients with HRS-AKI might have another indication for TIPS (‘pre-emptive TIPS’, failure of secondary prophylaxis, and recurrent/refractory ascites) [176]. (D2)
- Patients with HRS-NAKI should be evaluated for TIPS, since TIPS improves both renal function and survival in patients with severe/refractory ascites [98, 103]. (B1)

### Role of RRT and ELS in HRS-AKI and HRS-NAKI

- There are no randomized controlled trials demonstrating that renal replacement therapy (RRT) or extracorporeal liver support (ELS) improve survival in patients with HRS-AKI and HRS-NAKI, or associated conditions, such as acute-on-chronic liver failure (ACLF) [177, 178]. (B1)
- RRT should be evaluated in patients with treatment-refractory severe acidosis, electrolyte disturbances, or volume overload. (D1)
- Generally, RRT should be restricted to patients who are eligible for liver transplantation. However, even in this setting, there is no evidence of a survival benefit. (B1)
- A limited trial of RRT may be considered in selected non-liver transplant candidates, even though mortality rates are extremely high in patients not listed for liver transplantation [179]. (D2)
- In the absence of head-to-head comparisons, the optimal modality of RRT is unclear. However, continuous RRT use may be advantageous in patients who are hemodynamically unstable or at risk of elevated intracranial pressure (e.g., ACLF) [180].
- Regional citrate anticoagulation seems to be safe in patients with liver dysfunction, however, close monitoring for citrate accumulation is required [181]. (C1)

### Role of liver transplantation in HRS-AKI and HRS-NAKI

- Due to its poor prognosis, the diagnosis of HRS-AKI or HRS-NAKI should prompt evaluation for liver transplantation, which provides considerable benefit in this patient population, regardless of response to vasoconstrictor treatment [153]. (A1)
- However, it is still hard to predict to what extent renal failure is reversible after LT given potential pre-existing comorbidities, unrecognized intrinsic renal disease, intraoperative events, and post-transplant immunosuppression. Therefore, the indication for a simultaneous liver kidney transplantation (SLK) is still debated. Notably, transplantation of the liver first with the potential of performing a sequential kidney transplantation is the preferred option for patients with HRS-AKI according to the Eurotransplant manual (version 6.3) [182]. (C2)
- In contrast, EASL clinical practice guidelines recommend considering SLK transplantation in patients with cirrhosis and known significant kidney disease prior to HRS-AKI. Furthermore, SLK transplantation may be considered in patients with HRS-AKI on RRT or with an eGFR ≤ 35 mL/min or measured GFR ≤ 25 mL/min for ≥ 4 weeks [61]. (D2)

## 11. Transjugular intrahepatic portosystemic shunt (TIPS): evaluation, technical aspects, and follow-up

### Evaluation for elective TIPS placement

- Evaluation for elective TIPS placement requires more careful patient assessment and greater scrutiny towards contraindications, in particular in patients with the alternative option of timely liver transplantation; however, listing for liver transplantation does **not** preclude elective TIPS placement. (B2)
- In patients with contraindications for/refusing to undergo liver transplantation, the risk/benefit assessment and evaluation of resource utilization will allow a slightly more generous approach in favor of TIPS. (B2)
- The majority of elective TIPS placements will happen for recurrent or refractory ascites. This mandates careful evaluation of alternative reasons and important cofactors for ascites, which might not be improved or even worsened by TIPS placement. In particular, this relates to active infection (mostly SBP), malignant ascites, heart failure, and chronic kidney disease besides HRS-CKD. (B1)

### Evaluation for acute (‘pre-emptive’ or ‘early’ and ‘rescue’) TIPS placement

- ‘Pre-emptive’ (previously ‘early’) TIPS placement in patients without failure to control bleeding (i.e., if bleeding is controlled by vasoactive treatment and/or endoscopy) is only indicated for AVB in high-risk situations [183–185]. (B1) While evidence for pre-emptive TIPS placement is more robust in patients bleeding from esophageal varices and GOV1, this treatment concept may be similarly beneficial in those with GOV2/IGV1 [186]. (C1)
- High-risk patients are those with Child-Pugh C10–C13, Child-Pugh B8–B9 [185] and active bleeding at endoscopy under vasoactive treatment, or HVPG ≥ 20 mm Hg [184]. (B1)
- Pre-emptive TIPS should be placed preferably within 72 h (ideally 24 h) hours in order to prevent rebleeding and ACLF [183]. Due to logistic and time constraints, the pre-TIPS investigations may be limited to those necessary for confirming technical feasibility and for ruling-out absolute contraindications. (B1)
- This applies even more for rescue TIPS, i.e., TIPS placement in the context of failure to control bleeding (i.e., requirement of SEMS placement, balloon tamponade, or rebleeding within 5 days). (B1)
- In patients with high model for end-stage liver disease (> 30 points) and lactic acidosis (> 12 mmol/L), TIPS placement may be futile [187]. (C2)

### Investigations suggested prior to TIPS placement

- Four-phase CT of the liver and splenoportal axis. (D1)
- Laboratory investigation: Complete blood count, sodium, bilirubin, albumin, creatinine, AST/ALT, LDH, CRP, INR, and NT-proBNP, as well as lactate in unstable patients. (D1)
- Echocardiogram with a focus on right ventricular function, tricuspid regurgitation velocity (TRV), and/or estimated systolic pulmonal artery pressure (sPAP), and significant valvular heart disease. (B1)
- Right heart catheterization is suggested for those with a TRV > 2.8 m/s or systolic pulmonary artery pressure (sPAP) > 35 mm Hg, while it is required for elective TIPS placement in those with a TRV > 3.4 m/s or sPAP > 50 mm Hg to rule-out/evaluate the severity of (porto)pulmonary hypertension [188]. (B1)
- Ascites: See **Chap. 1 and 10**.

### Absolute contraindications for TIPS placement

- Severe liver failure (Child-Pugh ≥ C14) for elective TIPS placement. (B1)
- Severe and uncontrolled (porto)pulmonary hypertension (e.g., mean pulmonal artery pressure > 45 mm Hg) [189]. (D1)
- Symptomatic heart failure (in particular right heart failure). (B1)

### Relative contraindications for TIPS placement

- Anatomical/technical considerations, unrelieved biliary obstruction, or extensive (hepatic) malignancy. (B1)
- PVT or splanchnic vein thrombosis are *per se* is **not** a contraindication, but may even strengthen the rationale for TIPS placement, although it increases technical complexity of TIPS placement [190]. Patients requiring portal vein recanalization (PVR)-TIPS should be referred to expert centers. (B1)
- Bilirubin of > 3–5 mg/dL for elective procedures [190], while hyperbilirubinemia in the context of AD/ACLF is **not** a contraindication for early/rescue TIPS [191]. (C2)
- Recurrent overt HE episodes that are not related to acute bleeding, diuretic overuse, electrolyte disturbances, or infections. (C2)
- Asymptomatic (porto)pulmonary hypertension [189]. (B1)
- Asymptomatic heart failure. (B1)
- Any severe extrahepatic disease associated with a very limited life expectancy. (B1)

### Technical aspects of transjugular intrahepatic portosystemic shunt (TIPS) placement

There are different possibilities of TIPS placement, which basically differentiate systems with open Colapitano or Ross needles and systems with softer, closed coaxial needles where a stylette is advanced through a cannula [176]. In addition to the common and frequently practiced blind puncture with control of the portal vein incision via aspiration and direct contrast-enhanced visualization of the portal vein system, puncture towards the portal vein can also be guided by means of US or CO_2_ portogram. Such guidance systems for TIPS placement have been shown to reduce intervention times, radiation doses, and complication rates compared with conventional methods and provide additional information about the anatomical conditions during the intervention [192–199].

In the following, two techniques for TIPS placement are presented as examples. In the first case, US control using an open needle puncture system is shown, and in the second case, control by means of CO_2_ portogram using a closed coaxial needle puncture system is shortly pointed out. Besides blind puncture, the options shown below represent variants. In general, various approaches with both of the needle puncture systems and combinations of the single steps are possible, also depending on the anatomical conditions.

#### Example 1: Technique with open puncture system and US guidance

- US before the procedure with the patient already positioned: Extracorporeal marking of the portal vein bifurcation.
- Access via right internal jugular vein, if possible.
- Advance a stiff guidewire into the inferior vena cava through a short 10F airlock.
- Insertion of the possibly adapted pre-bent open Colapinto or Ross TIPS needle into the inferior vena cava with the tip safely retracted into the guiding catheter.
- Direct probing of the right hepatic vein (in exceptional cases the middle hepatic vein) with guiding catheter/needle system via the Amplatz wire and US control of the position plus digital subtraction angiography (DSA).
- Positioning the TIPS guiding catheter at the site identified by US and DSA for parenchymal incision.
- Liver parenchymal incision with the needle usually directed anteromedially under alternating control of ultrasound and fluoroscopy.
- Blood aspiration and, if necessary, injection with saline or, in the case of presumed location of the needle tip within the portal vein, with contrast medium for radiographic visualization of the regular portal incision. If correctly positioned, insertion of the Amplatz wire via the portal vein into the splenic vein or the superior mesenteric vein.
- Retraction of the guiding catheter/needle system.
- Exchange for a long 10F airlock.
- Insertion of a catheter (multipurpose or pigtail) over the Amplatz wire for DSA portogram and measurement of the PPG (gradient between the portal—measured via the catheter—and the hepatic vein/inferior vena cava—measured via the side-port of the sheath; notably right atrial pressure should not be used as a reference point [2, 21]) before TIPS placement. (C1)
- Pre-dilatation of the parenchymal tract with 8/80 mm balloon catheter.
- Stent graft placement: Covered stent graft (e.g., 10 mm GORE VIATORR) with the length of the covered portion selected according to the length of the parenchymal tract. If possible, the covered portion of the stent should not overlap the outlet of portal vein branches on the portal side. Moreover, the uncovered outermost distal portion of the stent (2 cm) should not reach into the parenchymal tract due to increased thrombogenicity. On the side of the hepatic vein, attention should be paid to a harmonically curved outflow tract in the area of the proximal stent end—otherwise, overlapping stent extension should be considered.
- TIPS expansion during the initial procedure: For ascites: 8 mm; for variceal bleeding indication 8 (to max. 10) mm aiming at achieving a target PPG of < 12 mm Hg or a > 50% reduction in patients with high pre-TIPS PPG values. (B1)
- Insertion of a catheter (multipurpose or pigtail) over the Amplatz wire for portogram and measurement of the PPG after each TIPS expansion in case multiple expansions are needed.
- In patients with bleeding, consideration of embolization of portosystemic collaterals in the gastric and esophageal regions that are still visualized.
- Removal of the devices and airlock and application of a light pressure bandage or exchange for a Quinton catheter.
- PPG values before TIPS placement and after each dilatation step should be stated on the report.

#### Example 2: Technique with closed puncture system and CO_2_ portogram

- As an alternative to the technique listed above, TIPS placement can also be performed with a more flexible closed coaxial puncture system and guidance via wedge CO_2_ portogram.
- If the hepatic veins are at a horizontal angle to the inferior vena cava, such systems may be particularly helpful.
- Imaging of the portal vein by CO_2_ portogram for puncture guidance is particularly useful in patients who are difficult to examine by US.

### Follow-up after TIPS placement

- Vasoactive drugs and conventional NSBBs/carvedilol can be discontinued after successful TIPS placement. (B1)
- Body weight should be monitored closely and timely diuretic dose reduction is recommended. (B1)
- In particular in patients with bleeding as the indication for TIPS placement, protocol imaging (Doppler ultrasound; flow velocities < 90 or > 190 cm/s are indicative of stenosis) is advisable before discharge as well as 3 and 6 months post TIPS placement and every 6 months thereafter, combined with HCC surveillance [200]. (C2)
- Additional ultrasound controls should be performed, if TIPS dysfunction is suspected due to lack/loss of clinical efficacy. (B1)
- Angiographic controls with hemodynamic evaluation are not routinely required but should be performed when there is a suspicion of TIPS stenosis/occlusion or dysfunction. Ideally, these angiographic controls are performed with the option of TIPS revision within the same session. (C2)
- In case of recurrent/persistent overt HE after TIPS, portosystemic pressure gradient (PPG) measurement should be performed. TIPS reduction/occlusion should be evaluated based on PPG and clinical status. (B1)

## 12. Portal vein thrombosis (PVT) in ACLD

In patients with an initial diagnosis of portal vein thrombosis (PVT), it is important to distinguish between ACLD-related PVT (common) and non-cirrhotic PVT (uncommon), since work-up and treatment are different. This consensus will only focus on PVT in patients with ACLD [201, 202]. (D1).

Severity of hepatic dysfunction and of portal hypertension, reduced portal vein velocity (< 15 cm/sec), and NASH aetiology increase the risk for PVT, which may also be triggered by local factors such as pancreatitis, infection, surgery, or trauma [203–205]. (D2).

### Screening and diagnosis

- Surveillance for PVT is recommended in all patients with ACLD, especially when evaluated or listed for liver transplantation, and is usually combined with HCC surveillance [2, 202, 206]. (B2)
- Screening for PVT should also be performed in patients with ACLD and new-onset or worsening of manifestations of portal hypertension, i.e., occurrence/worsening of ascites or bleeding from varices. Furthermore, PVT should be considered as a potential cause of abdominal pain in patients with ACLD [207]. (D1)
- Colour Doppler ultrasound is the first-line imaging method used to screen for PVT, despite lower sensitivity for partial thrombosis as well as technical limitations such as for detection of thrombosis of the portal trunk posterior to the duodenum and the superior mesenteric vein [2, 202, 205, 207, 208]. In case of uncertainty, contrast-enhanced cross-sectional imaging (CT scan or MRI imaging) should be performed. (B1)
- In patients with PVT, extension should always be evaluated by contrast-enhanced cross-sectional imaging. (B1)
- The role of inherited and acquired prothrombotic disorders for PVT development in patients with ACLD is unclear. Therefore, screening for underlying thrombophilia is not generally recommended. However, indication for screening should be decided on a case-by-case basis in patients with a family history of thrombosis, thrombosis at unusual sites, and a prior history of venous thromboembolism (VTE) [202, 207]. (D1)

### Characterization of a newly diagnosed PVT

- In order to enable subsequent evaluation of the spontaneous course and/or treatment indication, standardised documentation in the radiological report should include [209] (D1):
  - the extension within the splanchnic venous system,
  - the degree of luminal obstruction (complete/partial with ≥ 50% or < 50% of the original lumen occluded) in each individual vessel, and
  - chronicity of clot formation.
- In patients with an initial diagnosis of PVT, careful evaluation for associated hepatic malignancy is essential [202]. (C1)

### Prognosis following PVT development in ACLD

- PVT increases surgical complexity of liver transplantation and may have a negative impact on post-transplant outcomes [210, 211]. (B1)
- The impact of PVT on outcomes in non-transplant candidates with ACLD is still a matter of debate. While PVT development seems to be a symptom rather than a driver of clinical disease progression, (response) to anticoagulation was associated with a better outcome in retrospective studies [212–216]. Furthermore, PVT in patients with ACLD experiencing acute variceal bleeding (AVB) was associated with a higher risk of failure to control bleeding, rebleeding and short-term mortality [217, 218]. (C1)

### Indications for anticoagulation

- Patients with ACLD and PVT should receive anticoagulation in the following scenarios [2]: (C1)
  - Recent (< 6 months) completely or partially occlusive (> 50%) thrombosis of the portal vein trunk with or without extension to the superior mesenteric vein,
  - symptomatic PVT independently of the extension, or
  - any PVT in patients being candidates or listed for liver transplantation.
- Furthermore, anticoagulation should be considered in the following scenarios [2] (C2):
  - Minimally occlusive (< 50%) PVT of the portal vein trunk that progresses on short-term follow-up (1–3 months) or
  - compromises the superior mesenteric vein (SMV).

### Duration of anticoagulation and choice of drug

- Anticoagulation should be continued until transplantation in patients listed for or potential candidates for liver transplantation [2]. (C1)
- In non-liver transplant candidates, anticoagulation should be maintained until portal vein recanalization or for a minimum of 6 months [4, 16]. (C1)
- As recurrence of thrombosis frequently occurs, long-term anticoagulation may be considered in all patients achieving recanalization after individual risk/benefit-evaluation [12, 16]. (C2)
- Low molecular weight heparin (LMWH) may be used to initiate anticoagulation in patients with ACLD. LMWH and vitamin K antagonists (VKA) can be used for long-term anticoagulation in patients with ACLD [2, 212]. (C1)
- Anti-Xa monitoring of LMWH is **not** representative in patients with cirrhosis [219]. (C2)
- VKA should be monitored in patients with cirrhosis with an INR aimed at 2–3 [202]. (C1)
- Even though data on safety and efficacy is still limited, direct oral anticoagulants (DOACs) may be considered for long-term anticoagulation in patients with compensated cirrhosis (Child-Pugh class A). (C1)
- DOACs should only be used with caution in patients with Child-Pugh class B. (C2)
- DOACs should **not** be used in patients with severe hepatic impairment (Child-Pugh class C) [220–223]. (C2)
- Use of unfractionated heparin (UFH) should be restricted to special situations (e.g., eGFR < 30 ml/min or pending invasive procedures) due to the increased risk of heparin-induced thrombocytopenia [2]. (D2)
- In patients with an indication for medical treatment, anticoagulation should be started as early as possible, since early initiation of anticoagulation was found to be the most important factor predicting recanalization [224]. (C1)

### Follow-up evaluation

- Follow-up imaging should be performed with the same imaging technique after 3–6 months in patients undergoing anticoagulation [2]. (B1)
- First imaging follow-up in patients without current indication for anticoagulation should be performed within 4–6 weeks to monitor the course of disease [2, 207]. Further imaging schedule should be decided on a case-by-case basis. (C1)

### Prevention of bleeding events

- An adequate prophylaxis for gastrointestinal bleeding must be implemented prior to starting anticoagulation [2, 202]. (B1)
- Treatment with NSBBs may be preferred over endoscopic band ligation for primary prevention of variceal bleeding in patients with ACLD and PVT [225]. (D1)
- Patients with severe thrombocytopenia (e.g., < 50 G/L) are at higher risk of PVT, but also of bleeding complications on anticoagulation, thus, the risk/benefit ratio should be evaluated on a case-by-case basis [226, 227]. (C2)
- In patients at risk of falls (e.g., due to HE), risks and benefits should be evaluated on a case-by base basis [225]. (D1)
- Interruption of anticoagulant treatment for endoscopic band ligation does not seem to be mandatory, as there is data from small studies using LMWH/VKA suggesting that it can be safely performed [228–230]. Notably, even if interrupted, post-banding ulcer bleeding would usually occur after the restart of anticoagulation [231]. (C2)

### TIPS implantation for PVT in ACLD patients

- In order to improve post-transplant outcomes, evaluation for TIPS is recommended in patients listed or potential candidates for liver transplantation with thrombosis of the portal vein trunk without complete recanalization after 6 months of anticoagulation or with contraindication to anticoagulation [2, 232]. (C1)
- TIPS implantation should be considered in patients with ACLD and PVT experiencing severe portal hypertension-related complications [233]. (C2)
- The presence of portal cavernoma, no identifiable intrahepatic portal trunk or intrahepatic portal vein branches, and no appropriate landing zone increase technical difficulty of TIPS implantation, thus, requiring a careful risk/benefit assessment at an expert center [218]. (D2)
- No general recommendation regarding continuation or stopping of anticoagulant treatment after TIPS implantation for PVT can be made. The decision should consider risk/benefit assessment and post-TIPS flow [234]. (D2)

### PVT in ACLD patients with hepatic malignancies

- Patients with hepatic malignancies including hepatocellular carcinoma (HCC) are at high risk for developing malignant and non-malignant PVT [235, 236]. (B1)
- Occurrence of PVT in the presence of HCC does **not** directly imply vascular invasion. Malignant PVT is best diagnosed by triphasic CT or colour Doppler ultrasound/contrast-enhanced ultrasound and characterized by neovascularization of the thrombus, arterial enhancement with rapid washout and direct invasion by an adjacent hepatic mass [3, 236]. (B1)
- In general, anticoagulation is **not** indicated for malignant PVT, even though it may be considered in selected patients with symptomatic or progressive PVT [202]. (C2)
